# The prevalence of abdominal obesity and hypertension amongst adults in Ogbomoso, Nigeria

**DOI:** 10.4102/phcfm.v3i1.188

**Published:** 2011-03-08

**Authors:** Isaac O. Amole, Akintayo D. OlaOlorun, Louis O. Odeigah, Stephen A. Adesina

**Affiliations:** 1 Department of Family Medicine, Bowen University Teaching Hospital, Nigeria; 2 Department of Family Medicine, University of Ilorin Teaching Hospital, Nigeria

## Abstract

**Background:**

In many developing countries obesity and obesity-related morbidity are now becoming a problem of increasing importance. Obesity is associated with a number of disease conditions, including hypertension, type 2 diabetes mellitus, cardiovascular diseases, cancer, gallstones, respiratory system problems and sleep apnoea.

**Objectives:**

The aim of this study was to determine the prevalence of hypertension and obesity, as classified according to waist circumference (WC), and further to determine whether there was any association between abdominal obesity and hypertension amongst adults attending the Baptist Medical Centre, Ogbomoso, Nigeria.

**Method:**

A cross-sectional descriptive study of 400 adults aged 18 years or older was conducted. Blood pressure and WC measurements were taken and participants completed a standardised questionnaire.

**Results:**

A group of 400 participants were randomly selected (221 women; 179 men), with a mean age of 48.7 ± 16.6 years. The overall prevalence of obesity as indicated by WC was 33.8% (men = 8.9%; women = 53.8%). Women were significantly more sedentary than men (50.8% for men vs 62.4% for women, *p* < 0.05). Most of the obese participants’ families also preferred high-energy foods (85.2%, *p* > 0.05). Overall prevalence of hypertension amongst the study population was 50.5%, but without a significant difference between men and women (52.0% for men vs 49.3% for women, *p* > 0.05). The prevalence of hypertension amongst the obese subset, however, was 60.0%.

**Conclusion:**

Prevalence of abdominal obesity was found to be particularly significant amongst women in this setting and was associated with hypertension, physical inactivity and the consumption of high-energy diets.

## Introduction

In many developing countries obesity and obesity-related morbidity are becoming a problem of increasing importance.^1^ Urbanisation and economic development have led to a nutritional transition characterised by a shift to diets of higher energy content and/or to the reduction of physical activity, resulting in changes in individuals’ body composition.^1^ About 1.2 billion people worldwide are overweight and at least 300 million of them are obese.^2^ The World Health Organization (WHO) projects that more than 700 million adults worldwide will be obese by 2015.^3^

Obesity is defined as a condition of abnormal or excessive fat accumulation in the adipose tissue of the body.^4^ Body mass index (BMI), expressed as the ratio between weight (measured in kilogram) and the square of height (in metres), is used to measure the ‘degree of fatness’. A BMI between 25 and 29.9 is defined as overweight, whilst a value above or equals 30 is defined as obese.^4^ Normal weight is characterised by a BMI of between 18 and 24.9. A shortcoming of the BMI is that it does not give information about the total fat or fat distribution in the body, which can also affect health. For instance, fat deposited around the abdomen, especially in males, is far more dangerous than fat deposited in other parts of the body because abdominal fat is metabolically active while fat elsewhere may not be.^5^ The benchmark for assessment of abdominal adiposity is the use of imaging techniques, but in large epidemiological studies these methods are impractical because they are arduous and expensive.^5^ Waist circumference (WC), however, is considered a good anthropometric alternative for assessing abdominal adiposity. WC is an aggregate measurement of the actual amount of total and abdominal fat accumulation and is a crucial correlate of metabolic syndromes found amongst obese and overweight patients.^5^ Abdominal overweight is associated with a WC of 94 cm for men–101 cm for men and 80 cm for women–87 cm for women, whilest abdominal obesity is associated with a WC measurement exceeding 102 cm (for men) or 88 cm (for women).^4^ The major drawback of using WC to assess abdominal adiposity is that even with a standard protocol, clinicians’ measurements may differ.

Obesity is associated with a number of disease conditions, including hypertension, type 2 diabetes mellitus, cardiovascular diseases, cancer, gallstones, respiratory system problems and sleep apnoea.^6, 7^ According to the WHO, up to 20% of the population in developed countries may suffer from obesity-associated hypertension, which may account for 78% and 65% of essential hypertension in men and women, respectively.^8^ Hypertension is generally associated with a systolic blood pressure exceeding or equals 140 mmHg or a diastolic blood pressure of 90 mmHg or higher.^9^ Systolic and diastolic blood pressures are used to categorise normal blood pressure, pre-hypertension, stage 1 hypertension and stage 2 hypertension (Table 1).

**TABLE 1:** Blood pressure categories for adults (JNC-7)^9^.

BP classification	SBP (mmHg)	DBP (mmHg)
Normal	< or 120	< or 80
Pre-hypertension	120-139	80-89
Stage 1 hypertension	140-159	90-99
Stage 2 hypertension	≥ 160	≥ 100

*Source*: JNC-7 report BP, blood pressure; DBP, diastolic blood pressure; SBP, systolic blood pressure

### Setting

Ogbomoso is located about 100 km north of Ibadan, capital of the Oyo State in south-west Nigeria. The indigenous people belong to the Yoruba ethnic group, who engage mostly in farming or trading. There are two renowned academic institutions in Ogbomoso (Ladoke Akintola University of Technology and the Nigerian Baptist Theological Seminary), which attract people from other ethnic groups to the city. A government-owned general hospital, a Baptist mission hospital, a few primary health care centres and an increasing number of private hospitals meet the health needs of the people.

## Ethical considerations

The Ethics Committee of the Baptist Medical Centre, Ogbomoso granted approval for the study. Informed consent was also obtained from the participants before commencement of the study.

## Method

The study was conducted at the outpatients clinic of the Baptist Medical Centre, Ogbomoso between January and July 2008. The hospital is a 200-bed mission hospital that renders primary and secondary health care. It is the referral centre for all other hospitals in Ogbomoso. The aim of this study was to determine the prevalence of hypertension and obesity, as classified according to WC, and whether any association exists between them.

A cross-sectional descriptive survey was used. Consenting participants aged 18 years or older were recruited for the study. Pregnant women, women in the puerperium (up to 6 weeks post-delivery) and patients with ascites or intrabdominal masses (determined through history and physical examination) were excluded from the study.

A systematic sampling method was used to select the participants. The list of patients with appointments at the outpatients clinic was taken as a sample frame, and from a review of records an average of 100 patients were estimated to attend the clinic per day during the period of the study. A sampling fraction of 10 was chosen and according to a simple random sampling method the first subject from every 10 patients on the register was selected to participate. An identification sticker was placed on all selected participants’ record cards at the records office and sent to a consulting office designated for the study. The selected subjects were screened and those who met the inclusion criteria were recruited for the study upon obtaining informed consent. The identification sticker remained on each participant's card until completion of the study to avoid repeat selection. This sampling method yielded 400 participants aged 18 years or older.

Demographic information (age, gender, marital status, ethnic group, religion, nationality, occupation, educational status, physical activity, family history of hypertension and family eating habits) was obtained through a pre-tested questionnaire.

WC (in centimetres) was measured using a flexible, non-stretchable tape measure, at the midpoint between the lower rib border and the iliac crest at the end of expiration while participants were standing upright.^10^ Abdominal overweight was defined as a WC between 94 cm and 101 cm for men and between 80 cm and 87 cm for women, while abdominal obesity was defined as a WC ≥ 102 cm and ≥ 88 cm for men and women, respectively.^4^

Blood pressure was measured using an Accoson Dekamet mercury sphygmomanometer (Accoson, Essex) with an appropriate cuff size and a Littmann stethoscope (3M, Brookings). Blood pressure was measured in the right arm after at least 15 min of rest and while participants were sitting down.^11^ The cuff (about 12.5 cm wide) was applied evenly and snugly around the bare arm, with the lower edge 2.5 cm above the antecubital fossa. A thigh cuff (about 15 cm wide) was used for obese subjects. The cuff was inflated rapidly to about 30 mmHg above the level at which the radial pulse was no longer palpable, followed by slow deflation. The investigator listened with a stethoscope placed over the brachial artery in the antecubital fossa while deflating the cuff. Three readings, at least 2 min apart, were taken for each subject and the mean of the second and third readings were used for analysis.^11^ The systolic pressure was taken as the first-phase sound of Korotkoff and the diastolic pressure was taken as the fifth-phase sound of Korotkoff. The observed value was recorded to the nearest 2 mmHg. Hypertension was noted if systolic blood pressure exceeded 140 mmHg or diastolic blood pressure exceeded 90 mmHg,^9^ or upon self-report of a medical diagnosis of hypertension or current treatment for hypertension with prescription medication.^12^ Participants who engaged in 30 minutes’ leisure-time physical activity (walking, fitness training or sports) at least three times per week were classified as physically active.^13^

Participants were classified with regard to social class based on their occupation, as per the Registrar General's scale of social classes:^14^

- Class 1: Professional, for example lawyers, doctors, accountants
- Class 2: Intermediate, for example teachers, nurses, managers
- Class 3N: Skilled non-manual, for example typists, shop assistants, telephone operators
- Class 3M: Skilled manual, for example mineworkers, bus drivers, cooks, artisans
- Class 4: Partly skilled (manual), for example farm workers, bus conductors
- Class 5: Unskilled, for example cleaners, labourers.

Data were analysed with the statistical package for social sciences, version 13. (SPSS 13).

## Results

The mean age of the sample was 48.7 ± 16.6 years and there were more female (55.3%) than male participants (44.8%). Table 2 shows that the overall prevalence of obesity as determined according to WC was 33.8%. The prevalence of stage 1 and stage 2 hypertension was 23.5% and 20.5%, respectively, while the prevalence of pre-hypertension was 40.8%.

**TABLE 2:** Prevalence of abdominal obesity and hypertension.

Prevelence	Frequency	Percentage
**Abdominal obesity**		
Normal	194	48.5
Overweight	71	17.8
Obese	135	33.8
**Hypertension**		
Normal	61	15.2
Pre-hypertension	163	40.8
Stage 1	94	23.5
Stage 2	82	20.5

The association between abdominal adiposity, hypertension and demographic and lifestyle variables is shown in table 3. Obesity increased with age, but was most prevalent amongst the age group 40-49 years. The prevalence of obesity amongst men was 8.9% and 53.8% amongst women (*p <* 0.05), and almost two-thirds (64.4%) of obese participants were physically inactive (*p >* 0.05). The overwhelming majority of obese participants’ families (85.2%) preferred to consume starchy foods (*p* > 0.05) although the majority of the obese participants (71.9%) indicated to eat fast food only rarely (*p* > 0.05). More than half the obese participants (62.2%) were from social class 3N (*p* < 0.05). Hypertension increased with age and the prevalence of hypertension amongst the study population was 50.5%. The prevalence of hypertension amongst the obese participants was 60.0%. The prevalence of stage 1 and stage 2 hypertension amongst the obese participants was 30.4% and 23.0%, respectively.

**TABLE 3:** Association between abdominal adiposity, gender, family menu, social class and hypertension.

Variables	Abdominal adiposity								*χ*^*2*^	*p-*value

	Normal		Overweight		Obese (%)		Total (%)			
				
	*n*	%	*n*	%	*n*	%	*n*	%		
**Age range**									64.825	0
18–19	5	100.0	0	0.0	0	0.0	5	1.3	-	-
20–29	47	83.9	6	10.7	3	5.4	56	14.0	-	-
30–39	34	51.5	12	18.2	20	30.3	66	16.5	-	-
40–49	20	27.8	11	15.3	41	56.9	72	18.0	-	-
50–59	27	32.5	19	22.9	37	44.6	83	20.7	-	-
60–69	35	48.6	16	22.2	21	29.2	72	18.0	-	-
≥70	27	58.7	6	13.0	13	28.3	46	11.5	-	-
**Gender**									117.72	0
Male	139	77.7	24	13.4	16	8.9	179	44.8	-	-
Female	56	25.4	46	20.8	119	53.8	221	55.2	-	-
**Family menu**										
Starchy	174	89.2	65	92.9	115	85.2	354	88.5	-	-
Fat	0	0.0	0	0.0	0	0.0	0	0.0	-	-
**Total**	**195**	**100.0**	**70**	**100.0**	**135**	**100.0**	**400**	**100.0**	5.874	0.209
**Social class**										
Class 1	3	1.5	1	1.4	0	0.0	4	1.0	-	-
Class 2	37	19.0	18	25.7	32	23.7	87	21.8	-	-
Class 3N	53	27.2	34	48.6	84	62.2	171	42.7	-	-
Class 3M	15	7.7	4	5.7	3	2.2	22	5.5	-	-
Class 4	43	22.1	4	5.7	3	2.2	50	12.5	-	-
Class 5	44	22.6	9	12.9	13	9.6	66	16.5	-	-
**Total**	**195**	**100.0**	**70**	**100.0**	**135**	**100.0**	**400**	**100.0**	68.401	0
**Hypertension**										
Hypertensive	85	43.6	36	51.4	81	60.0	202	50.5	-	-
Normotensive	110	56.4	34	48.6	54	40.0	198	49.5	-	-
**Total**	**195**	**100.0**	**70**	**100.0**	**135**	**100.0**	**400**	**100.0**	8.623	0.013
**Hypertension grade**										
Normal	32	16.5	15	21.1	14	10.3	61	15.2	-	-
Pre-hypertension	86	44.3	28	39.4	49	36.3	163	40.8	-	-
Stage 1	40	20.6	13	18.4	41	30.4	94	23.5	-	-
Stage 2	36	18.6	15	21.1	31	23.0	82	20.5	-	-
**Total**	**194**	**100.0**	**71**	**100.0**	**135**	**100.0**	**400**	**100.0**	10.192	0.117
**Fast food**										
Rarely	135	69.2	47	67.1	97	71.9	279	69.8	-	-
Occasionally	58	29.7	21	30.0	36	26.7	115	28.7	-	-
Very often	2	1.1	2	2.9	2	1.4	6	1.5	-	-
**Total**	**195**	**100.0**	**70**	**100.0**	**135**	**100.0**	**400**	**100.0**	1.622	0.805
**Physical activity**										
Active	93	47.9	30	42.3	48	35.6	171	42.7	-	-
Inactive	101	52.1	41	57.7	87	64.4	229	57.3	-	-
**Total**	**194**	**100.0**	**71**	**100.0**	**135**	**100.0**	**400**	**100.0**	4.996	0.082

*X*^*2*^, Chi-square; *n*, Given as means of number

Study variables are summarised according to gender in Tables 4 and 5. The mean WC amongst males was 84.1 ± 12.7 cm and 90.4 ± 14.6 cm amongst females (87.6 ± 14.1 cm over the entire group). The prevalence of physical inactivity amongst the participants was 57.3% (62.4% for females and 50.8% for males, *p* < 0.05).

**TABLE 4:** Gender, physical activity and mean values for age and waist circumference.

Physical activity	Male		Female		Total		χ^*2*^	*p-*value
		
	*n*	%	*n*	%	*N*	%		
Active	88	49.2	83	37.6	171	42.7	-	-
Inactive	91	50.8	138	62.4	229	57.3	-	-
Total	179	100.0	221	100.0	400	100.0	4.442	0.02
**Variables**								
Age (years)	49.3 ± 18.1		48.1 ± 15.3		48.7±16.7		-	-
WC (cm)†	84.1 ± 12.7		90.4 ± 14.6		87.6±14.1		-	-

*χ*^*2*^, Chi- square

† WC (cm), waist circumference; *n*, Given as means of number; *n*, Given as means of total number.

The prevalence of hypertension amongst female participants was 49.3% compared to 52.0% amongst males (*p* > 0.05) as shown in table 5.

**TABLE 5:** Association between hypertension, age and gender

Variables	Hypertensive		Normotensive		*χ*^*2*^	*p*-value
	
	*n*	(%)	*n*	(%)		
**Age range**					**51.217**	**0**
18–19	1	20.0	2	80.0	-	-
20–29	12	21.4	44	78.6	-	-
30–39	20	30.3	46	69.7	-	-
40–49	41	56.9	31	43.1	-	-
50–59	47	56.6	36	43.4	-	-
60–69	56	63.9	26	36.1	-	-
≥ 70	35	76.1	11	23.9		
**Gender**					**0.274**	**0.6**
Male	93	52.0	86	48.0	-	-
Female	109	49.3	112	50.7	-	-

*χ*^*2*^, Chi- square; *n*, Given as means of number

## Discussion

WC measurements from this study show that obesity increased with age, but peaked amongst participants between 40-years and 49 years of age. This is similar to the finding of Siminialayi, Emem-Chioma and Dapper^15^ in Rivers State, Nigeria, which showed that abdominal obesity was more common amongst subjects older than 40 years. The prevalence of obesity as determined according to WC measurements (33.8%) is comparable to results from Okrika, Rivers State, Nigeria^15^ (31.7%) and Cotonou, Benin Republic^16^ (32.0%). Rguibi and Belahsen,^17^ however, found the prevalence of abdominal obesity amongst women in Sahraoui, Morocco to be 76.0%, which is higher than what was found in the present study amongst the women (53.8%).

The high prevalence of physical inactivity amongst women in this study may be one of the factors that could be responsible for their high prevalence of obesity. In a study regarding the association between measures and determinants of obesity in African women, Kruger, Venter, Vorster and Margetts^18^ found that physical inactivity showed the strongest association with measures of obesity. Their findings are in line with those of Afolabi, Addo and Sonibare,^19^ who conducted a study in Abeokuta, Ogun State, Nigeria. In addition, consumption of high-energy diets is one of the major contributing factors to the development of obesity, as underlined by our finding that the majority of obese participants’ families (85.2%) preferred starchy food. However, contrary to the findings of Fezue, Minkoulou, Balkau, et al.^20^ where obesity was strongly associated with high socio-economic status, more than half (62.2%) of the participants classified as obese here fell in social class 3N. This observation may be due to low representation of participants from social class 1 in this study.

This study also showed that the prevalence of hypertension increases with age, with participants aged 70 years or older showing the highest prevalence (76.1%) of hypertension (*p* < 0.05). This is not surprising because it has been established that age is a predisposing factor for the development of essential hypertension.^21^Our findings are in line with those of Olatunbosun, Kaufman, Cooper and Bella,^21^ who found that age is a risk factor for the development of hypertension in the urban black population in Ibadan, Nigeria. The overall prevalence of hypertension amongst our study population was 50.5%, which may be attributed to the specific setting; hypertension is one of the leading disease entities at the Baptist Medical Centre. The prevalence is, however, higher than in earlier studies in Ghana and elsewhere in Nigeria, where the prevalence of hypertension was found to be between 10.3% and 26.8%.^22, 23, 24^ The fact that these studies were community based may be one of the reasons for observing lower overall prevalence of hypertension. Using different cut-off points for hypertension may also have contributed to the different prevalence rates of hypertension observed in earlier studies. Olatunbosun et al.^21^ defined hypertension as systolic or diastolic blood pressure ≥ 160/95 mmHg, while the cut-off in this study was ≥ 140/90 mmHg. The prevalence of pre-hypertension (40.8%) was much higher than that of stage 1 (23.5%) and stage 2 (20.0%) hypertension. Several studies have shown lifestyle modification, which is the main treatment modality in pre-hypertension, to be effective at preventing hypertension and lowering the risk of blood pressure-related clinical complications in the whole population.^9, 25^ Therefore, lifestyle modification needs to be promoted amongst patients at Ogbomoso in order to prevent participants who presented with pre-hypertension from progressing to stage 1 or stage 2.

The higher prevalence of hypertension in men despite a much higher rate of abdominal obesity in women is similar to the findings of Olatunbosun et al.^21^ in Ibadan, Nigeria. This finding is not surprising in a Black population because it has been documented that men are predisposed to developing hypertension in most Black populations.^21^

The study showed that hypertension was strongly associated with obesity in Ogbomoso, although the symptoms suggest mostly pre-hypertension, rather than having progressed already to stage 1 or stage 2.

## Conclusion

The findings from this study suggest that abdominal obesity in patients presenting at this health care facility in Ogbomoso is particularly significant amongst women and is associated with hypertension, physical inactivity and the consumption of high-energy diets. Measurement of WC requires only the use of a tape measure and therefore most clinics in developing countries should be able to make WC measurement a routine procedure, rather than calculating BMIs, to screen for obesity. This will help to identify patients at risk of developing obesity early and instituting measures such as physical exercise that will lead to a reduction in waist sizes before complications associated with obesity can develop.

Because patients are likely to be already familiar with their waist sizes, it will be easier for them to learn how to measure and interpret their WC, rather than calculating a BMI, of which interpretation is likely to be an abstract concept. People who appreciate the dangers associated with large waists can then institute measures such as increasing physical exercise and adjusting their diet to get their waists back to normal, and thus reduce their risks of disease conditions associated with obesity.
